# Rapid identification of extensively and extremely drug resistant tuberculosis from multidrug resistant strains; using PCR-RFLP and PCR-SSCP

**Published:** 2012-12

**Authors:** P Tahmasebi, P Farnia, FM Sheikholslami, AA Velayati

**Affiliations:** Mycobacteriology Research Centre, National Research Institute of Tuberculosis and Lung Disease (NRITLD), WHO & UNION Collaborating Centre for TB & Lung Diseases, Darabad, Tehran, Iran

**Keywords:** *Mycobacterium tuberculosis*, ciprofloxacin, amikacin, PCR-RFLP, PCR-SSCP

## Abstract

**Background and Objectives:**

Resistance in *Mycobacterium tuberculosis* is caused by mutations in genes encoding drug targets. Investigators have already demonstrated the existence of mutations in codons 88 to 94 in the *gyrA* gene and also in codons 1400, 1401, and 1483 of *rrs* gene among extensively and extremely drug resistant tuberculosis (XDR & XXDR-TB) strains. The aim of this study was to identify the XDR and XXDR-TB stains based on their mutational analysis.

**Materials and Methods:**

Susceptibility testing against first and second–line anti-tuberculosis drugs was performed by the proportional method. Based on susceptibility results, samples were later analyzed, using PCR-SSCP and PCR-RFLP for detection of mutation in *gyrA* and *rrs* genes.

**Results:**

Overall, using proportional method, sixty-three strains (64.9%) were identified as MDR, 8(8.2%) as non-MDR and 26 strains (26.8%) were susceptible. Thirty-one cases (31.9%) were amikacin-resistant and 18 (18.5%) samples were ciprofloxacin-resistant. Using PCR-SSCP and PCR-RFLP, we identified 6(6.2%) and 7(7.2%) resistant strains, respectively. Discrepancy in strains was cross-checked by sequencing. The results showed no mutation in 66.6% and 77.4% of CIP and AMK- resistant strains.

**Conclusion:**

Rapid detection of drug-resistant *Mycobacterium tuberculosis* using molecular techniques could be effective in determining therapeutic regimen and preventing the spread of XDR and MDR TB in the community. We should still keep in mind that a high number of resistant strains may have no mutation in proposed candidate genes.

## INTRODUCTION

The emergence of extensively drug resistant strains (XDR- TB) among patients with multidrug resistant (MDR-TB) tuberculosis was reported in different continents. XDR-TB is defined as an MDR strain which shows resistance to fluoroquinolone and to any of the three injectable drugs (amikacin, caperomycin or kanamycin). More recently, a new dangerous form of resistant tuberculosis bacilli was identified. This group of strains showed *in-vitro* resistance to the entire first and second–line drugs tested. They were named as totally drug resistant (TDR) or extremely drug resistant (XXDR-TB) strains (1–5). The patients infected with XDR and XXDR-TB are difficult to treat and may increase the risk of disease transmission among the community. Clinically, ciprofloxacin and amikacin are recommended for the treatment of MDR-TB patients (6). Ciprofloxacin (CIP) is the synthetic derivative of nalidixic acid, classified as a fluoroquinolone (FQ). FQs bind with DNA gyrase (heterotetramer composed of two A and two B subunits, encoded by the *gyrA* and *gyrB* genes, respectively) and inactivate it (7). The quinolone-resistance determining region (QRDR) of the *gyrA* and *gyrB* genes is the conserved region and mutation in this region is responsible for resistance to FQs. Mutation were frequently reported at codon 88 to 94 of the *gyrA* gene, however, a less frequently mutation was also seen at codon 495, 516, 533 of *gyrB* gene (8). Resistance to amikacin (AMK) is associated with nucleotide change at positions 1400 (substitution A to G) 1401 (substitution C to A) and 1483 (substitution G to T) in the rrs gene (that encoding *16s rRNA*) (9). At present, detection of drug resistance is performed by proportional methods. It takes at least 6 to 12 weeks to determine the susceptibility patterns. In the present study, we tried to identify the MDR and XDR-TB isolates using molecular techniques, i.e., PCR-RFLP (PCR-Restriction fragment length polymorphism) and PCR-SSCP (PCR-Single-strand conformation polymorphism). Thereafter, the results were compared with sequencing and classical susceptibility testing.

## MATERIALS AND METHODS

### Bacterial susceptibility testing

Samples were collected from patients with a history of TB treatment failure referred to Mycobacteriology Research Center, Masih Daneshvari hospital, Tehran. Primary isolation and culturing of Mycobacterium isolates from sputum specimen was done in accordance with standard solid-culture procedures (10). All isolates (n = 97) were identified as *M. tuberculosis* by biochemical tests, including niacin production, catalase activity, nitrate reduction, pigment production and growth rate (10). Drug susceptibility tests against isoniazid (INH), rifampicin (RF), streptomycin (SM), ethambutol (ETB) were performed by the proportional method on Löwenstein-Jensen media at a concentration of 0.2, 40, 4.0 and 2.0 µg/ml, respectively. Drug-susceptibility test against second–line drugs (kanamycin, amikacin, caperomycin, ciprofloxacin, cycloserine, ethionamide and para-aminosalicylic acid) was performed using two critical proportions of 1% and 10% (11). Isolates used in this study were collected from patients clinically and laboratory diagnosed as susceptible (n = 26), MDR (n = 63 isolates), Non-MDR(n = 8) and XDR TB(n = 13) cases.

### DNA extraction and amplification

DNAs for PCR were prepared by CTAB method described previously (12).

### PCR for SSCP

To amplify a 320 bp region of *gyrA*, primers gyrA-F (5′ -CAGCTA CATCGACTATGCGA -3′) and gyrA-R (5′- GGGCTTCGGTGTACCTCAT -3′) were used (13). PCR mixture (25 µl) contained: 10 X PCR buffer (2.5 µl); 1.5 m M MgCl2; DMSO4%; 0.4 mM dNTP; 0.32 mM (each) primer; and 2. 5 U of HotStar Taq^®^ Plus DNA polymerase (Qiagen, Germany); 10 ng DNA Template.

The reaction mixtures were then put in the thermal cycler (Astec, Japan) that carried out the following PCR programs: (1) 95°C for 10 minutes, 95°C, 60 °C and 72°C (1 minute each), for 2 cycles, 95°C, 59°C and 72°C (1 minute each), for 2 cycles, 95°C, 58°C and 72°C (1 minute each), for 2 cycles, 95°C, 57°C and 72°C (1 minute each), for 2 cycles, 95°C, 57°C and 72°C (1 minute each), for 35 cycles with a final 7 minutes extension step at 72°C.

After amplifying, PCR products were electrophoresed with a suitable size marker on a 1.5% agarose gel. Then, PCR products were purified by PCR purification kit (Fermentas, Germany).

A 7µl of purified PCR product was mixed with 7 ul of gel loading buffer (95% formamide, 0.05% bromophenol blue, 0.05% xylene cyanol, 20 mM EDTA), heated for 10 min at 94°C, cooled on ice, and loaded onto a nondenaturing gel composed of: 10 ml of 30% acrylamid sulotion, 160 µl of freshly 10% ammonium persulfate, 2.4 ml of 10x TBE buffer, 2.5 ml of glycerol, 16 µl of TEMED, up to 40 ml volume. Electrophoresis was performed at room temperature overnight at 105 V. Afterwards, single strand bands were observed with silver staining.

### PCR for RFLP

To amplify a 460 bp region of *rrs*, primers rrs 1096 (5′- GCGCAA CCCTTGTCTCATGTTG -3′) and rrs1539 (5′- GGGGCGTTTTGCTGGTGCTCC -3′) were used (14). PCR mixture for 50 µl reaction consist of: 10 X PCR buffer (5 µl), 1.5 mM Mgcl_2_, 0.2 mM from each deoxynucleotide triphospates (dNTP), 0.2 mM (each) primer, 2 µl DMSO, 1.25 U HotStar Taq^®^ Plus DNA polymerase (Qiagen, Germany), 10 ng DNA Template. PCR program was performed as follow: 95°C for 10 minutes, 95°C, 60 °C and 72°C (1 minute each), for 40 cycles with a final 10 minutes extension step at 72°C. The PCR products were observed on a 1.5% agarose gel. Amplified products digested with the restriction endonucleases *Tai*I (Fermentas, Germany) and *Dde*I (Roche, Germany) according to the manufacturer's procedures. Then, digested PCR products were separated by electrophoresis on a 8% poly acrylamid gel (Table 1).

**Table 1:** Length of digested fragments by restriction endonucleases *TaiI* and *DdeI*.

	Digested Fragments by *Dde*I	Digested Fragments by *Tai* I
Susceptible Strains	248,191,22 bp	187,154,,59,31,30 bp
Resistant Strains	196,191,52,22 bp	187,185,59,30 bp

To confirm the results of molecular techniques, DNA sequencing was performed (Gene Fanavaran Company, Iran).

## RESULTS

The proportional method identified 63 isolates (64.9%) as MDR-TB strains. The remaining strains were either susceptible (26.8%) or detected other resistance patterns (non MDR; 8; 8.2%). Among MDR-TB strains, 13.4% were XDR and 6.1% were TDR-TB isolates. Overall, our results showed 31 and 18 amikacin- resistant strains and CIP- resistant strains, respectively.

### Detection of CIP-resistant strains by PCR-SSCP Analysis

Using PCR with touch-down programs all strains produced a strong band of 320 bp of *gyrA* gene PCR products. PCR-SSCP showed that, 6 specimens (6.2%) as resistance to CIP and 90 specimens (93.8%) was susceptible. The SSCP patterns of *gyrA* for CIP- resistance *M. tuberculosis* isolates were clearly differentiated from that of the susceptible strains based on the migration pattern of a single strand of DNA through the polyacrylamide gel. We found 7 different SSCP patterns (2 among susceptible strains and 5 among CIP-resistant isolates), and the identities of the mutations were characterized by DNA sequencing (Table 2).

**Table 2:** Mutations in the QRDR of *gyrA*.

Codon with *gyrA* mutation, amino acid (nucleotide) change:	No. (%) of strains	

	Resistant to CIP	Susceptible to CIP
No mutation	12 (66.6)	79 (81.5)
90, Ala (GCG)→Val (GTG)	1 (5.5)	
94, Asp (GAC)→Gly (GGC)	1	
94, Asp (GAC)→Asn (AAC)	2 (11.1)	
94, Asp (GAC)→Tyr (TAC)	1	
94 & 91		
91, Ser (TCG)→Pro (CCG)		
&	1	
94, Asp (GAC)→Asn (AAC)		
Total	18 (18.5)	79 (81.5)

**Fig. 1:**
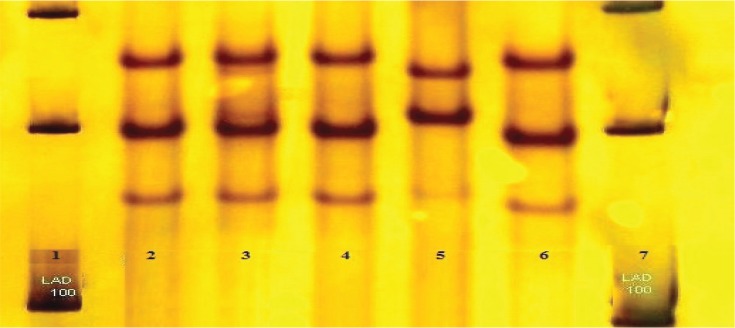
Susceptible SSCP patterns on SSCP gel. Lane 2,3,4,6 polymorphism pattern (had mutation in coden 95), lane 5 pattern of standard strain H37Rv (had no any mutation), lane 1,7 DNA maker 100 bp plus.

**Fig. 2:**
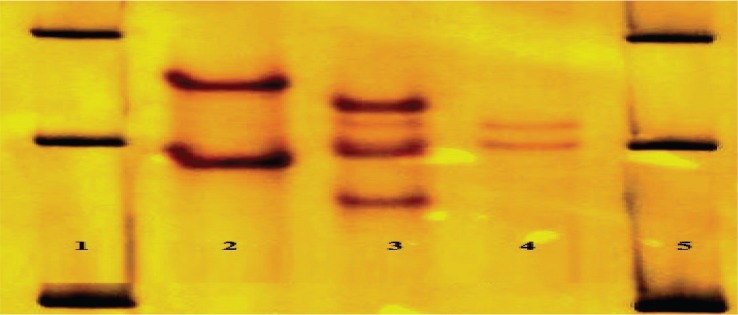
Resistant SSCP patterns on SSCP gel. Lane 2 mutant Tyr-94, lane 3 mutant 91 & 94, lane 4 mutant Val-90, lane 1,5 DNA maker 100 bp plus.

**Fig. 3:**
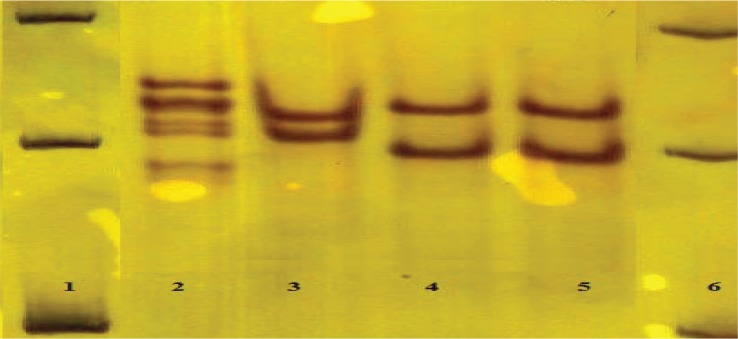
Resistant SSCP patterns on SSCP gel. Lane 2 mutant Asn -94, lane 3 mutant Gly-94, lane 4, 5 mutatant 95 (susceptible pattern), lane 1,6 DNA marker 100 bp plus.

Five different resistant SSCP patterns were produced by mutation in 90, 94 codons and double mutation in 91 and 94 codons. The most frequent mutation detected was the substitution at codon 94 (4/6 strains [66.6%]). AT codon 90, substitution C to T, which led to an amino acid change of alanine to valine was found in one strain. Also, double mutation in codon 91 and 94 was found in one strain (Fig. 1–3). All resistant strains have mutation in codon 95.

Two different susceptible SSCP patterns were observed: one in strains with mutation in codon 95 and the other in those strains with no mutation (Fig. 1). In addition, in 12(66.6%) of CIP- resistant strains no mutation associated with resistant was found.

### Detection of amikacin-resistant strains by PCR-RFLP analysis

**Fig. 4:**
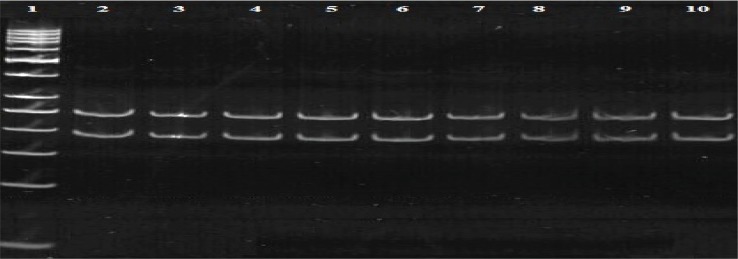
PCR products digested by *Dde* I on 8% polyacrylamide gel. 2-9 is the sensitive pattern (248, 191 bp), lane 10 H37RV(standard strian has no mutation). lane1, 50 bp DNA marker.

**Fig. 5:**
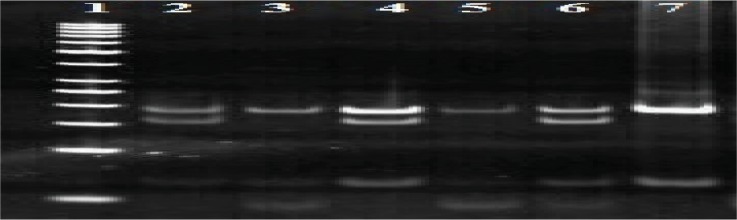
PCR products digested by *TaiI* on 8% polyacrylamide gel. Lane 3, 5, 7 is the resistant pattern(185, 187 bp), lane 2, 4 is the sensitive pattern (187, 154 bp), lane 6 H37RV (standard strian has no mutation). lane 1, 50 bp DNA marker.

After amplifying the 460 bp of the *rrs* gene, PCR products were electrophoresed with a suitable size marker on a 1.5% agarose gel. Digestion with *Dde*I enzyme has shown the sensitive pattern (191, 248 bp) in all 97 isolates. That means no mutation at 1483 codon was observed (Fig. 4). Digestion with *Tai*I has shown resistance pattern in 7 specimens (185, 187 bp) and 90 (92.8%) specimens have shown sensitive pattern (154, 187 bp) (Fig. 5). This result showed that 7(7.2%) specimens were resistant to amikacin and the remaining were susceptible.

### DNA sequencing

The PCR products were used as a template for the sequencing reaction, using the primers gyrA-F (5′-CAGCTACATCGACTATGCGA-3′) for the sense strand and gyrA-R (5′- GGGCTTCGGTGTACCTCAT -3′) for the reverse strand. Also for *rrs* gene sequencing rrs1096 (5′-GCGCAACCCTTGTCTCATGTTG-3′) and rrs1539 (5′-GGGGCGTTTTGCTGGTGCTCC -3′) primers were used. Mutations in *gyrA* and *rrs* were identified by comparing them with the *M. tuberculosis* strain H37Rv sequence (standard strain that have no any mutation). The results of molecular techniques were confirmed by sequencing.

## DISCUSSION

In order to prevent the spread of drug-resistant *M. tuberculosis* and prescribe an effective treatment regimen for patients with drug-resistant tuberculosis, use of rapid molecular methods are necessary. In the present study in which PCR-SSCP was used, 93.8% of strains were susceptible and 6.2% were resistant to CIP. Seven SSCP patterns were identified and the identities of the mutations were established by DNA sequencing. The resistant strains had five different SSCP patterns, whereas in susceptible strains only two different SSCP patterns were observed. Our findings of mutations in the *gyrA* gene are similar to those reported from other parts of the world, especially the common mutations, which reflects a global pattern (8, 13, 15–17). The results also show that the frequency of mutation in codon 94 (66.6%) of the *gyrA* gene was higher compared to other codons. The high substitutions at position Asp94 in fluoroquinolone-resistant strains of *M. tuberculosis* was reported by other researchers (8, 13, 18). The high frequency of mutation in codon 94, leads us to consider this codon as a candidate marker for fluoroquinolone resistance detection and a possible indicator of an XDR case (19). All CIP-resistant strains have mutation in *gyrA* codon 95. Mutation at this codon is not associated with FQ resistance, but it seems to initiate other mutations associated with FQ resistance.

Furthermore, we observed no mutation in QRDR of 12 (66.6%) CIP-resistant strains. The finding is in agreement with previous reports in which no mutations were found in this region (8, 18–20). This strain possibly carries a mutation which is located outside the QRDRs, or the resistance may be caused by other mechanisms. In separate studies by Sullivan *et al*.(1995) and Kocagoz *et al*., (1996), it was proposed that only 42–85% of fluoroquinolone resistance isolates might have *gyrA* mutations in the QRDR region (20, 21). Thus, by using molecular technique there is a chance of missing such strains. That may be a reason for many reference TB Laboratories to use other rapid–detection systems, in addition to molecular detection. These results were confirmed using DNA sequencing; all isolates (100%) with PCR-SSCP resistant patterns, showed mutation by DNA sequencing. The CIP sensitive strains have either no mutation in QRDR or have no mutation in codon 95 *gyrA*.

Based on PCR-RRLP, 7.2% of isolates had AMK resistance and the remaining isolates were susceptible. The frequency of mutation in *rrs* gene was higher at codon 1400 than other codons. In this regard, Taniguchi (1997) and Suzuki (1998) proposed that nucleotide substitutions at codon 1400 of *rrs* gene may be used as an important marker of high-level AMK-KAN resistance (9, 22). Although the 1483 position of *16S rRNA* would be a site for resistance mutations, but it occurs at a lower frequency (7). Isolates with intermediate susceptibility to AMK and KAN show no mutations in the rrs (9, 22). The resistant isolates which had no mutation in the QRDR of *gyrA* or in *rrs* gene, probably carries a mutation which is situated outside this regions, or the resistance may be caused by other mechanisms, such as enhanced drug efflux (23), or morphological changes may help them escape the immune response and/or overcome the inhibitory effect of most antibiotics (3). On the other hands 16 isolates from 17 MDR–TB strains (17.52%) that were sensitive to second-line drugs, could be correctly detected (94 .1%) using molecular techniques. Rapid and accurate detection of MDR cases that were sensitive to second-line drugs is important for effective and accurate treatment.

PCR-SSCP and PCR-RFLP are rapid and simple techniques which can be used as an additional method to detect resistant cases, but these methods have their own limitations in action. Standardization of PCR- SSCP is difficult and some factors such as: fragment size, temperature during electrophoresis, concentration of acrylamide/bisacrylamide, and glycerol concentration can affect the mobility of single-strand DNA. Therefore, results have been hard to repeat in other laboratories. In addition, not all mutations result in the gain or loss of a restriction site. Broad use of RFLP to screen mutations associated with drug resistance is limited (24). Finally, as resistance to AMK and CIP plays a central role in identifying XDR-TB, it is increasingly important to establish rapid molecular tests to detect resistance to these drugs.
